# Supplementary figures and images for: Expression analysis of genes and pathways associated with liver metastases of the uveal melanoma
Source: BMC Med Genet. 2014 Mar 5;15:29. doi: 10.1186/1471-2350-15-29 (PMC4015751; doi:10.1186/1471-2350-15-29)

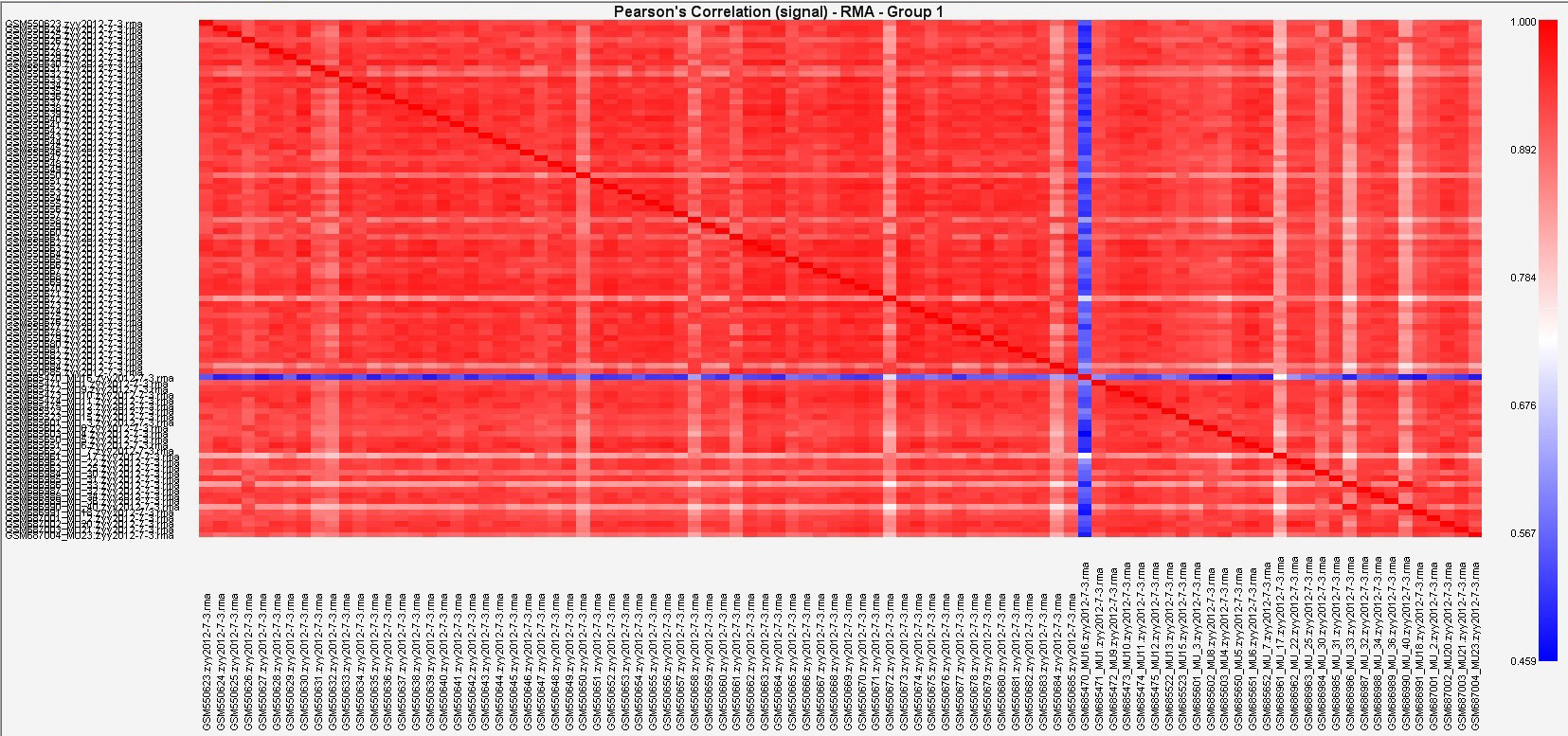

Supplement: Additional file 1: Figure S1 — Normalized microarray data by Pearson’s Correlation. Uveal melanoma samples microarray data were normalized by Pearson’s Correlation and one sample GSM685470 was removed. [file 1471-2350-15-29-S1.tiff]

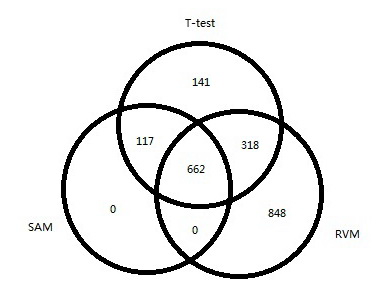

Supplement: Additional file 2: Figure S2 — Venn diagram for different methods of gene expression analysis. T-test, SAM and RVM mode were used to screen the differential expressed genes. [file 1471-2350-15-29-S2.tiff]
